# Frequency and determinants of thyroid autoimmunity in Ghanaian type 2 diabetes patients: a case-control study

**DOI:** 10.1186/s12902-016-0152-4

**Published:** 2017-01-17

**Authors:** Osei Sarfo-Kantanka, Fred Stephen Sarfo, Eunice Oparebea Ansah, Ernest Yorke, Josephine Akpalu, Bernard C. Nkum, Benjamin Eghan

**Affiliations:** 1 Directorate of Medicine, Komfo Anokye Teaching Hospital, Endocrine and Diabetes Unit, P.O. Box 1934, Kumasi, Ghana; 2 Department of Medicine, School of Medical Sciences, Kwame Nkrumah University of Science and Technology, Kumasi, Ghana; 3 Department of Medicine, University of Ghana School of Medicine and Dentistry, Accra, Ghana

**Keywords:** Thyroid autoimmunity, Type 2 diabetes mellitus, Associated factors

## Abstract

**Background:**

The link between type 1 diabetes and thyroid autoimmunity is well described. The same cannot be said for type 2 diabetes where results have been mixed so far. We investigated the prevalence and determinants of thyroid autoimmunity among Ghanaian type 2 diabetes patients.

**Methods:**

This was a case-control study involving 302 type 2 diabetes patients and 310 non - diabetic controls aged 40–80 years. Anthropometric and blood pressure measurements were obtained. Fasting samples were analyzed for glucose, thyroid function, and antibodies to thyroglobulin and thyroid peroxidase.

**Results:**

The prevalence of thyroid autoimmunity was significantly higher among T2DM subjects (12.2% vs. 3.9%, *p* = 0.0004). Among T2DM subjects, 44 (14.7%) tested positive for TPOAb, 5 (1.7%) tested positive for TGAb and 15 (5.0%) tested positive for both autoantibodies. Females T2DM subjects showed a 3-fold increased risk of thyroid autoimmunity compared to males (OR:3.16, *p* =0.004), T2DM subjects with hyperthyroidism had a 41% increased risk of thyroid autoimmunity (OR: 1.41, *p* < 0.001), sub-clinical hyperthyroidism increased the risk of thyroid autoimmunity by 2 fold, (OR:2.19, *p* < 0.001), subclinical hypothyroidism increased the risk of autoimmunity by 4-fold, (OR:3.57 95% *p* < 0.0001), and hypothyroidism was associated with a 61% increased risk of thyroid autoimmunity (OR: 1.61,1.35–2.23). Dyslipidaemia was associated with a 44% increased risk of thyroid autoimmunity (OR: 1.44, *p* = 0.01) and a percentage increase in HbA1c was associated with 46% increased risk of thyroid autoimmunity (OR:1.46, *p* < 0.0001).

**Conclusion:**

We observed a high prevalence of thyroid autoimmunity in Ghanaian T2DM subjects compared to the general population. Thyroid autoimmunity in T2DM subjects was significantly associated with female gender, thyroid dysfunction, dyslipidaemia and poor glycemic control.

**Electronic supplementary material:**

The online version of this article (doi:10.1186/s12902-016-0152-4) contains supplementary material, which is available to authorized users.

## Background

Diabetes and thyroid disorders represent the two commonest endocrinological conditions seen in adult medical practice [1, 2]. The concurrence of the two conditions in the same individual can prove inimical to achieving good glycemic control and further multiply the cardiovascular risk associated with diabetes. [1, 2] Studies worldwide have shown a higher prevalence of thyroid dysfunction in type 2 diabetes (T2DM) patients and vice-versa [2–8]. The spectrum of thyroid disorders (like diabetes) is wide; and it is continuously experiencing a change in epidemiology, usually determined by iodine levels seen in the population in focus [9, 10]. In arears of the world where intake of iodine, a major component of thyroid hormones, is sufficient, autoimmune disorders represent the commonest cause of thyroid pathology [11]. In contrast, there is widespread dietary iodine deficiency in Africa, which underlines most of the clinical and pathological presentations of thyroid disease [12]. Recently, with the remarkable improvement in iodine nutrition through widespread salt iodination on the continent, there appears to be a shift in thyroid epidemiology towards autoimmunity [13]. Thyroid autoimmunity which comprises a number of distinct but pathogenically related immune-mediated destructive disorders of the thyroid gland is often characterized by the presence of autoantibodies directed mostly against thyroid peroxidase (TPOAb) and thyroglobulin(TGAb) [14]. Type 1 diabetes has an established association with autoimmune thyroid disorders through a common genetic inheritance [15, 16]. Studies to investigate a link between thyroid autoimmunity and T2DM have produced mixed results so far, mostly beset by differing methodologies, iodine statuses and sensitivities of immunological tests employed in determining thyroid autoantibodies [17–21]. Whiles Akbar et al. [4] obtained a significantly higher prevalence of thyroid autoimmunity in the study of Saudi T2DM subjects, Afkhami- Ardekani et al. [22] study of Iranian T2DM subjects did not yield any significant difference between the two groups. Among Africans, there exist a gaping hole of literature documenting thyroid autoimmunity both among the general population and T2DM patients. Cardoso et al. in one of the few studies on the continent to date, compared type 1 diabetes patients and controls with T2DM subjects for thyroid autoantibodies and obtained a predictably low autoantibody level of 1.7% among T2DM subjects [23]. As far as we are aware, no recent published studies were cited on the topic on the continent to reflect the changing epidemiology of thyroid diseases among Africans toward increased autoimmunity. The aim of this study was therefore to determine the prevalence and the associated factors of thyroid autoimmunity in Ghanaian T2DM patients.

## Methods

This was a case-control study in which cases were consecutive patients with established T2DM defined by the WHO criteria [18], self-reported diagnosis of diabetes and/or treatment with antidiabetic medications (among patients who were insulin non-requiring in the first year after diagnosis for glycemic control). Cases were recruited from the outpatient diabetes clinic of Komfo Anokye Teaching Hospital (KATH), the second largest tertiary referral hospital in Ghana from April 2014 to April 2015. Community in-dwelling age and sex matched adults from the same region were recruited to serve as controls after normoglycemia was documented by both fasting plasma glucose (FPG) and glycated hemoglobin (HbA1c). Using a structured validated questionnaire and a review of medical records we obtained the sociodemographic and clinical information of all participants. Because of their confounding effects on thyroid function, we excluded pregnant women, patients on amiodarone, lithium and long-term corticosteroids as well as those with an acute illness and history of hospitalization less than 6 months from the day of recruitment.

### Ethical approval and consent to participate

The study was approved by the Committee for Human Research Publications and Ethics at the School of Medical Sciences, Kwame Nkrumah University of Science and Technology and the Komfo Anokye Teaching Hospital, Kumasi. All participants gave an informed consent with those unable to understand or sign the informed consent excluded.

### Study measurements

#### Physical measurements

Body weight and height were taken in duplicates using a combined manual scale and stadiometer (Asimed MB 211 T plus Asparatos Y Sistemas de Medida,). Body mass Index (BMI) was calculated as weight in kilogram divided by the square of height in meters (kg/m^2^). Overweight/general obesity was defined as BMI ≥25 kg/m^2^ [17].

### Waist circumference measurement

Duplicate waist circumference (WC) measurements were taken and the average recorded for both group of participants, WC measurements > 80 cm and 94 cm were recorded as central obesity for females and males respectively [16].

### Blood pressure measurement

Duplicate blood pressure recordings were taken with the participant in a seated upright position using a standard mercury sphygmomanometer after at least 15 min of rest. Hypertension was defined as mean blood pressure ≥ 140/90 mm Hg and/ or documented antihypertensive therapy [19].

Smokers were identified by self-report as those who had smoked at least 10 sticks of cigarette per day for 6 months or more or those who smoked daily for 1 year or more regardless of the number of cigarettes smoked per day [20]. Positive alcohol intake status was identified when greater than 14 units of alcohol was consumed per week in the case of a female and 21 units per week in case of a male [21].

### Laboratory measurements

Approximately ten milliliters (10mls) of fasting venous samples were collected from each participant into vacutainer tubes (Becton Dickinson, Rutherford, and N.J) and Sequestrene bottles. Samples were manually processed and cryopreserved before transporting to laboratory for analysis. Fasting plasma glucose (FPG), thyroid profile: free thyroxine (FT4), free triiodothyronine (FT3), thyroid stimulating hormone (TSH), total cholesterol (TC), low-density lipoprotein cholesterol (LDL-C), high-density lipoprotein cholesterol (HDL-C), triglycerides (TG)], urea, creatinine, TGAb and TPOAb were assayed by Chemoimmunoluminiscence method (Roche Diagnostics, Cobas e411 automated immunoassay analyzer, Indianapolis, USA) following the manufacturer’s instructions. Glycated hemoglobin (HbA1c) measurements were performed by standardized high performance liquid chromatography assay using Bio-Rad Variant II hemoglobin testing autoanalyzer. The reference range, intra-assay and interassay coefficients of variation for thyroid hormones and antibodies were as follows:

(TSH: O.25–5.0 IU/ml, <2.1% and <2.4%, FT3: 3.7–10.4pmol/l, 5.8% and 6.9% for FT3, FT4: 7.5–21.1pmol/l, 2.8% and 2.4%, TPOAb > 5.6 U/L, 2.1% and 6.1%, TGAb > 4.1U/L, 1.9% and 5.6%).

Thyroid function was classified as: Euthyroidism - when FT4, FT3 and TSH were within the normal range, hypothyroidism when TSH level was greater than the upper limit of the reference range and FT4/ or FT3 is lower than the lower limit of their reference ranges, Subclinical hypothyroidism- when TSH is greater than the upper limit of the reference range and FT4 and FT3 are within the normal range. Hyperthyroidism- when TSH level is lower than the upper limit of the reference range and FT4/or FT3 is greater than the upper limit of their reference ranges, subclinical hyperthyroidism-when TSH level is lower than the lower limit of the reference range and FT_3_ and FT_4_ are within the normal range. Thyroid autoimmunity was defined as positive TPOAb and/or TGAb.

Dyslipidemia was defined as TG level ≥3.0 mmol/L and HDL cholesterol level (<1.0 mmol/L) regardless of patient’s gender [24].

### Statistical analysis

Data was analyzed using Graph Pad Prism 7 software for Mac OS X. Continuous and dichotomous variables were presented as mean (standard deviation) and n (%) respectively. Data normality assumption was performed by visual inspection of distribution as well as D Agostino and Pearson Omnibus normality test. Statistical difference between means, medians and proportions were assessed using student t-tests, Mann-Whitney U tests and chi-square test respectively. To adjust for the effects of confounders, logistic regression models was carried out to identify independent predictors of thyroid autoimmunity. A significant level of *P* < 0.05 was used for the analysis.

## Results and discussion

### Baseline characteristics of T2DM subjects and controls

The overall study population was 612 (comprising 302 T2DM subjects and 310 controls). Table 1 describes the baseline characteristics of T2DM subjects and controls. There was no difference in age (57.6 ± 9.4 vs. 57.2 ± 9.5, *p* = 0.65) and percentage of females (58.9 vs 58.4, *p* = 0.95) between the 2 groups of participants. Type 2 diabetes subjects had significantly higher mean systolic blood pressure (148.2 ± 20.5 vs. 130.4 ± 22.7, *p* < 0.0001), mean diastolic blood pressure (82.6 ± 12.6 vs. 77.6 ± 12.7, *p* < 0.0001), median BMI [27.9 (21.5–31.5) vs. 27.0 (24–30.3, *p* = 0.03)], waist circumference [98 (90–106) vs. 94 (84–100.8) *p* < 0.0001] and proportion with dyslipidemia (53% vs. 11%, *p* < 0.0001) compared to controls. The proportion of participants that smoked or drank alcohol was not significantly different between the two groups.

**Table 1:** Demographic and Clinical Characteristics of Study participants

Variable	T2DM subjects	Controls	*P*- Value
Number	302	310	
Age, years (mean ± SD)	57.6 ± 9.4	57.2 ± 9.5	0.65
Hypertension, n(%)	213 (70.5)	75 (24.5)	<0.0001
Female gender, n (%)	178 (58.9)	181 (58.4)	0.95
SBP, mmHg (mean ± SD)	148.2 ± 20.5	130.4 ± 22.7	<0.0001
DBP, mmHg (Mean ± SD)	82.6 ± 12.6	77.6 ± 12.7	<0.0001
Dyslipidemia, n (%)	159 (52.6)	24 (11.4)	<0.0001
Alcohol, n (%)	87 (28.8)	120 (29.0)	0.96
Smoking, n (%)	22 (7.3)	15 (4.8)	0.24
Body mass index, kg/m^2^, median (IQR)	27.9 (21.5–31.5)	27.0 (24–30.3)	0.03
Waist Circumference, cm, median (IQR)	98 (90–106)	94 (84–100.8)	<0.0001
Central obesity, n (%)	210 (69.5)	121 (39.0)	<0.0001
HbA1c,%, median (IQR)	8.2 (6.9–9.6)	5.2 (5.0–5.4)	<0.0001
Fasting blood glucose, mmol/L, (Mean ± SD)	10.4 ± 5.4	5.3 ± 1.4	<0.0001
Creatinine,μmol/L, median (IQR)	84 (62–106.8)	77 (63–99)	0.21
Thyroid dysfunction, n (%)	56 (18.5)	40 (12.9)	0.06
Thyroid autoimmunity, n (%)	64 (21.2)	12 (3.9)	<0.0001
Positive TPOAb, n (%)	44 (14.7)	6 (1.9)	<0.0001
Positive TGAb, n (%)	5 (1.7)	3 (0.9)	0.99
Positive TPOAb + TGAb n (%)	15 (5.0)	3 (0.9)	0.004

### Prevalence of thyroid dysfunction and autoimmunity between the two groups

As shown in Table 1, the prevalence of thyroid dysfunction between the two groups was not significant (18.5 vs. 12.9, *p* = 0.06). The prevalence of thyroid autoimmunity among T2DM participants was about 5-fold higher than in controls (21% vs 4%, *p* < 0.0001). Of the 302 T2DM subjects, 14.7% (*n* = 44) tested positive for TPOAb, 1.7% (*n* = 5) for TGAb and 5% (*n* = 15) for both antibodies. For controls 2% (*n* = 6), 1% (*n* = 3) and 1% (*n* = 3) tested positive for TPOAb, TGAb and both antibodies respectively. Significant difference existed in the two groups in the prevalence of TPOAb (14.7% vs 1.9, *p* < 0.0001), TPOAb and TGAb (5.0% vs 0.9%, *p* = 0.004). The difference in prevalence of TGAb between the two groups was not significant (1.7% vs 0.9%, *p* = 0.95).

**Fig. 1:**
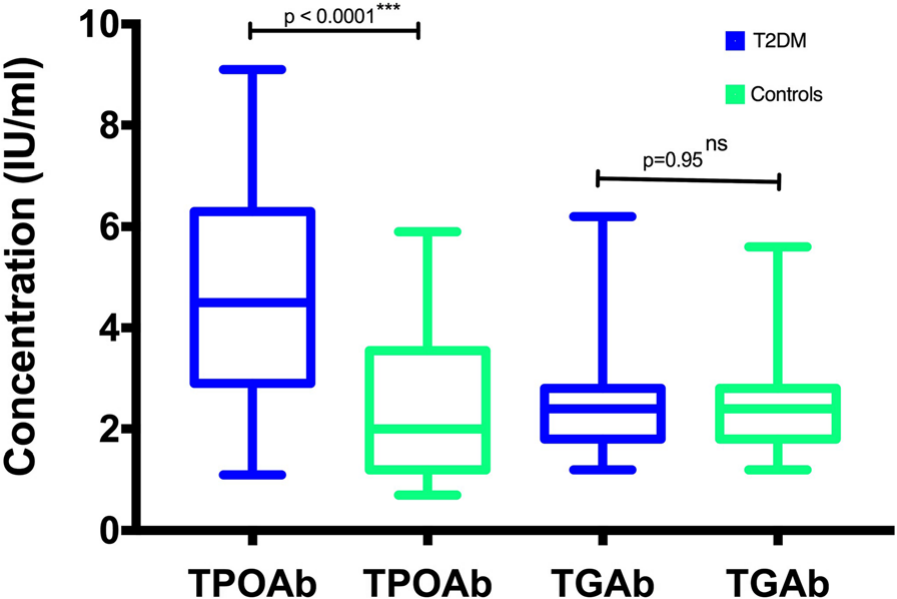
Mean concentration of thyroid autoantibodies in study participants

Figure 1 shows that the median concentration of TPOAb was significantly higher in T2DM patients compared to controls [4.5(2.9–6.3) vs 2.0(1.2–3.6), *p* < 0.0001]. The median concentration of TGAb was not significantly different between the groups [2.4 (1.8–2.8) vs 2.3 (1.6–2.8), *p* = 0.95].

### Frequency of thyroid thyroid dysfunction among autoimmunity positive participants

**Fig. 2:**
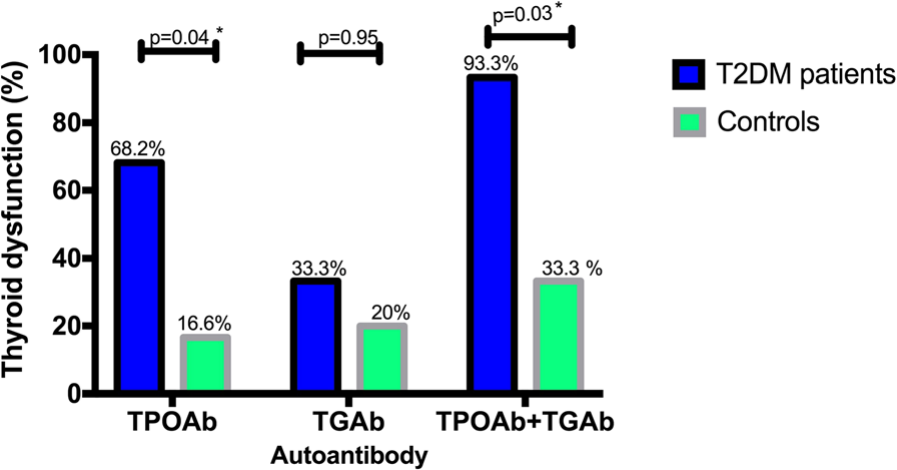
Thyroid dysfunction in autoantibody positive T2DM subjects and Controls

Figure 2 shows the frequency of thyroid dysfunction among autoimmune positive participants. Of the 44 T2DM subjects who tested positive for TPOAb, 68% (*n* = 30) had thyroid dysfunction, 20% (*n* = 1) of the 5 patients who tested positive for TGAb had thyroid dysfunction whiles 93% (*n* = 14) of the 15 patients with both antibodies had thyroid dysfunction. Among controls, 17% (*n* = 1) of TPOAb positive had thyroid dysfunction, 20% (*n* = 1) of those with TGAb and TPOAb had thyroid dysfunction. The presence of TPOAb and both antibodies was significantly associated with thyroid dysfunction in T2DM subjects compared to controls. The difference in the proportion of TGAb positive participants with thyroid dysfunction was not significant between the two groups.

### Clinical and laboratory characteristics of T2DM subjects according to autoantibody status

As shown in Table 2, T2DM subjects with thyroid autoimmunity had significantly higher FPG [9.7 (8.7–11.2 VS. 7.6 (6.7–9.4), *p* <0.0001], HbA1c [8.2 (6.8–9.8) vs. 6.1 (5.2–9.6) *p* <0.0001], TC [6.03 (4.7–7.06) vs. 4.9 (3.9–5.7), *p* < 0.0001], LDL-C [3.87 (2.83–5.08) vs. 2.90 (2.0–3.60), *p* < 0.0001] and TSH [3.4 (0.1–8.1) vs 1.1(0.8–1.8) *p* < 0.04] compared to T2DM subjects without thyroid autoimmunity. There was no significant difference between the two groups in terms of age, duration of diabetes, blood pressure and renal function.

**Table 2:** Characteristics of T2DM subjects according to thyroid autoimmunity status

Variable	Positive Thyroid autoimmunity	Negative thyroid autoimmunity	P
Number (%)	64 (21.2)	239 (78.8)	<0.0001
Female Gender, n (%)	58 (70)	138 (57.7)	0.04
Age, years, (mean ± SD)	57.5 ± 9.0	57.4 ± 9.5	0.93
Duration of T2DM, median (IQR)	5 (3–10)	5 (2–10)	0.49
Systolic blood pressure, median (IQR)	140 (130–160)	139 (130–150)	0.33
Diastolic blood pressure, median (IQR)	85.5 (70–90)	80 (72–90)	0.31
Smoking, n (%)	7 (13.0)	15 (6.3)	0.27
Alcohol, n (%)	31 (48.4)	86 (36.0)	0.08
BMI, Kg/m^2^, median (IQR)	24.7 (24.8–30.4)	26.9 (24.5–32.1)	0.79
Waist circumference, cm, median (IQR)	99 (88–109)	95 (86–102)	0.04
Fasting blood glucose, mmol/L, median (IQR)	9.7 (8.7–11.2)	7.6 (6.7–9.4)	<0.0001
HbA1c % median IQR	8.2 (6.8–9.8)	6.1 (5.2–9.6)	<0.001
TC median IQR	6.03 (4.7–7.06)	4.9 (3.9–5.7)	<0.001
LDL-C, mmol/L median IQR	3.87 (2.83–5.08)	2.90 (2.0–3.60)	<0.0001
HDL-C, mmol/L median (IQR)	1.22 (1.0–1.45)	1.18 (1.0–1.5)	0.41
Triglycerides, mmol/L, median (IQR)	1.25 (0.99-1.67)	1.19 (0.9-1.60)	0.18
Creatinine, mmol/L, median (IQR)	84 (62–106)	77 (63–99)	0.21
TSH, pmol/L, median (IQR)	3.4 (0.1–8.1)	1.1 (0.8–1.8)	<0.04

### Associations of thyroid autoimmunity in T2DM subjects

The results of multiple logistic regression analysis are shown in Table 3 were as follows; after adjusting for BMI, T2DM subjects with thyroid autoimmunity had a 3-fold increased risk of being females, (OR: 3.16 95% CI: 1.46–6.87, *p* < 0.0001), a percentage increase in HbA1c increased the odds of thyroid autoimmunity by 46% (OR: 1.46 95% CI 1.23–1.73) and a mmol increase in TC increased the odds of thyroid autoimmunity by 44%. The odds of thyroid dysfunction were increased in T2DM subjects’ with thyroid autoimmunity with a 2-fold increased odds of subclinical hyperthyroidism; (OR: 2.1 95% CI: 1.7–2.6, *p* < 0.0001), 1.41× increased odds of clinical hyperthyroidism (OR: 1.41: 95% CI: 1.2–1.98, *p* < 0.0001), 3.8× increased odds of subclinical hypothyroidism (OR: 3.8 95% CI: 2.7–3.8 *p* < 0.0001) and 61% increased odds of clinical hypothyroidism (OR: 1.61 95% CI :1.35–2.23, *p* < 0.0001).

**Table 3:** Multiple Logistic Regression Analysis for determinants of autoimmunity in T2DM patients

Variable	Unadjusted Odds ratio 95% CI	*p*-value	Adjusted Odds ratio 95% CI	*p*-value
Age				
For each 10 years older	1.00 (0.88–1.15)	0.96	-	-
Gender				
Female	4.45 (2.17–9.15)	<0.0001	3.16 (1.46–6.87)	0.004
Male	1.00			
Thyroid function				
Euthyroidism	1.00	-		
Clinical hyperthyroidism	1.98 (1.04–3.04)	<0.0001	1.41 (1.20–1.98)	<0.0001
Clinical hypothyroidism	2.21 (2.11–3.41)	<0.0001	1.61 (1.35–2.23)	<0.0001
Subclinical hyperthyroidism	2.5 (2.19–2.90)	<0.0001	2.19 (1.70–2.58)	<0.0001
Subclinical hypothyroidism	5.0 (4.60–5.50)	<0.0001	3.57 (2.74–3.82)	<0.0001
Glycaemic state				
For every 1% increase in HbA1c	1.83 (1.53–2.19)	<0.0001	1.46 (1.23–1.73)	<0.0001
Cholesterol status				
For 1 mmol increase in total Cholesterol	1.55 (1.28–1.87)	<0.0001	1.44 (1.17–1.77)	0.01

## Discussion

There are limited studies comparing autoantibody prevalence between T2DM subjects and controls worldwide. Our study has shown a higher prevalence of thyroid autoimmunity in Ghanaian T2DM subjects compared to controls with one in five T2DM subjects testing positive for thyroid autoimmunity compared to one in twenty seen among the controls. This finding is consistent with those of Akbar [4], Yasmin [25] and Konstantinos [26] who recorded significantly higher prevalence of thyroid autoimmunity in T2DM subjects compared to controls, with prevalence ranging between 10% and 43% among T2DM subjects. On the contrary, Cardoso et al. [4] and Afkhami- Ardekani et al. [22] recorded no significant difference between the 2 groups. The discrepancy in the results of studies investigating the prevalence of thyroid autoimmunity in T2DM subjects may be as a result of different methodologies employed in the determination of autoantibodies. It has been shown that the prevalence of these autoantibodies increases as the sensitivity of the assay method increase. This may have accounted for the highly significant increase in prevalence of autoantibody prevalence seen in our study. Cardoso employed manual ELISA methods whiles we used a more sensitive 2- site Chemiluminescent automated method in our determination [27]. Additionally, differences in case groups, especially in terms of differing ages, race and ethnicity, varying sample sizes, gender composition, geographic area, duration of diabetes of subjects in the individual studies may have accounted for the difference in results obtained. Although not tested in our study, it has been shown that T2DM subjects have reduced levels of Vitamin D [28, 29], a situation that can trigger autoimmunity and serve as a link between T2DM and thyroid autoimmunity as seen in our study.

Autoimmune disorders generally, including thyroid autoimmunity, are commonly associated with female gender compared to males due to the role of estrogen as an immunomodulator [29]. Additionally, there is an increased susceptibility of females to antibody formation in response to stress due to an increased T helper (Th) 2- predominant immune response compared to male where cytotoxic response is elicited from T helper (Th) 1 response [30]. Similarly, our study found that females T2DM subjects had a 3-fold increase risk of thyroid autoimmunity compared to males. Our study had a significantly higher representation of females though.

There was varying concentration of thyroid antibodies among T2DM subjects and controls with the median level of TPOAb significantly higher among T2DM subject compared to controls. With respect to TGAb there was no significant difference between the two groups. The higher levels of TPOAb may be due to the increased stimulation of thyroid autoantibody collaborating well with increased lymphocytic infiltration of the thyroid gland [31].

The presence of thyroid autoimmunity was significantly associated with subclinical thyroid disease with almost 3 fold increased risk of subclinical thyroid disease. Clinical thyroid disease including hyperthyroidism and hypothyroidism were also increased by about 1.5× fold in the presence of thyroid autoimmunity. This finding suggests that the presence of thyroid antibodies may serve as an indicator of both overt and subclinical thyroid dysfunction [32, 33]. Majority of those with subclinical disease may in the presence of thyroid autoantibodies expected to progress to overt thyroid disease as seen in participants of the Freemantle study [34].

In T2DM subjects with thyroid dysfunction, there is an increase insulin resistance usually manifesting as worsened lipid levels and poor glycemic controls [35, 36]. This is seen in our study where patient with thyroid autoimmunity most of which was associated with thyroid dysfunction had an almost 2-fold higher levels of glycated hemoglobin and dyslipidaemia. Additionally, it has been shown that thyroid autoimmunity correlate well with autoimmune destruction of beta- cells (though not a significant pathophysiology in T2DM), and this can lead to worsening of glycaemic control as seen in our study [37].

A major limitation of this study was our inability to test for Thyroid Stimulating Immunoglobulins which may indicated the cause of hyperthyroidism in some of the cases. Additionally, our inability to test for Glutamic Acid Decarboxylase Autoantibody type 65 (GAD 65) especially in T2DM subjects in the early forties meant we may have enrolled patients with Latent Autoimmune Diabetes of Adults in the study. Also to be noted is our inability to use oral glucose tolerance test in ruling out diabetes in our controls. With this, a marginal misclassification of patients with glucose intolerance may have been included as controls. Considering the observational nature of this evidence and, thus, the inappropriateness for causality inference, we advise caution in the interpretation of these findings. Especially in extrapolating these findings to different populations with different baseline characteristics. However, the impact of these limitations on our study findings is probably minimal, since the discrimination between the T2DM subjects and the control group was based on two glycemic indices (fasting glucose and HbA1c measurements), which secured a clear distinction between groups.

The strength of this study compared to a comparable study among West Africans is the increased sample size of 310 T2DM subjects compared to 60 patients in the first study. Future studies should be designed to study the influence of other factors including Vitamin D status on thyroid autoimmunity in T2DM subjects.

## Conclusion

The results of the present study indicate that the frequency of thyroid autoimmunity is significantly higher in Ghanaian T2DM patients, with its presence significantly associated with thyroid dysfunction, female gender, hypercholesterolemia and hyperglycemia. Therefore, it is necessary to screen type 2 diabetes patients especially females with thyroid dysfunction for thyroid autoimmunity.

## Additional file

Additional file 1: Excel File with Participant Information. (XLSX 125 kb)

## Abbreviations

BMI: Body mass index
FT3: Free triiodothyronine
FT4: Free thyroxine
HDL-C: High-density lipoprotein cholesterol
LDL-C: Low-density lipoprotein cholesterol
T2DM: Type 2 diabetes mellitus
TC: Total cholesterol
TG: Triglycerides
TGAb: Thyroglobulin autoantibody
TPOAb: Thyroid peroxidase autoantibody
WC: Waist circumference
